# Genomic insights into ceftazidime resistance in *Burkholderia pseudomallei*: discovery of A172T mutation and palindromic GC-rich repeat sequences facilitating *penA* duplication and amplification

**DOI:** 10.1128/aac.00220-25

**Published:** 2025-07-21

**Authors:** Apichai Tuanyok, Chie Nakajima, Tiernan Noll, Md. Siddiqur Rahman Khan, Pacharapong Khrongsee, Charles A. Yowell, Yu-Ping Xiao, Vanaporn Wuthiekanun, Narisara Chantratita, Henry Heine, Kuttichantran Subramaniam, Yasuhiko Suzuki, Direk Limmathurotsakul, Ayalew Mergia

**Affiliations:** 1 Department of Infectious Diseases and Immunology, College of Veterinary Medicine, University of Florida 828883, Gainesville, Florida, USA; 2 Emerging Pathogens Institute, University of Florida 145775, Gainesville, Florida, USA; 3 Hokkaido University International Institute for Zoonosis Control, Sapporo, Hokkaido, Japan; 4 Hokkaido University Institute for Vaccine Research and Development, Sapporo, Hokkaido, Japan; 5 Faculty of Veterinary Science, Prince of Songkla University 634604, Hat Yai, Songkhla, Thailand; 6 Mahidol–Oxford Tropical Medicine Research Unit, Faculty of Tropical Medicine, Mahidol University 115374, Bangkok, Thailand; 7 Department of Microbiology and Immunology, Faculty of Tropical Medicine, Mahidol University 115374, Bangkok, Thailand; 8 Institute for Therapeutic Innovation, Department of Medicine, College of Medicine, University of Florida 209735, Gainesville, Florida, USA; Universita degli Studi di Roma "La Sapienza", Rome, Italy

**Keywords:** *Burkholderia pseudomallei*, *penA*, ceftazidime, melioidosis, AMR

## Abstract

Ceftazidime (CAZ) resistance in *Burkholderia pseudomallei*, the causative agent of melioidosis, complicates treatment in endemic regions. This study identified a novel A172T mutation and other known *penA* mutations as critical contributors to CAZ resistance in a large Thai strain collection. Frequent gene duplication and amplification of *penA*, likely driven by Palindromic GC-Rich Repeat Sequences, highlights the urgent need for rapid diagnostics and optimized treatment strategies to manage this life-threatening disease effectively.

## INTRODUCTION

The growing threat of antimicrobial resistance (AMR) in *Burkholderia pseudomallei* demands a deeper understanding of the mechanisms underlying ceftazidime (CAZ) resistance. In this study, we analyzed a collection of 58 strains isolated from 24 patients who experienced treatment failures in northeast Thailand over two decades, 1987–2007. These strains were specifically chosen for further analyses due to their previously documented Etest results indicating resistance to CAZ and amoxicillin-clavulanic acid (AMC), which developed during or after treatment (1). Although Etest was previously used to determine the minimal inhibitory concentrations (MICs), it is not an endorsed method for *B. pseudomallei* by the Clinical and Laboratory Standards Institute (CLSI) or the European Committee on Antimicrobial Susceptibility Testing and thus represents a methodological limitation. To address this, we re-determined the MICs using the broth microdilution (BMD) technique, following CLSI guidelines (2). Resistance to CAZ and AMC was observed in the same isolates from these patients (Table 1). Details of these isolates and their MICs for both antibiotics from the previously reported Etest and the current BMD results are provided in Table S1. To identify the genetic factors contributing to this resistance, we employed next-generation sequencing on the Illumina MiSeq platform as previously described (3), followed by a detailed analysis of the sequencing data using the BWA-MEM algorithm (4). Genomic alignments were conducted against the reference genome of *B. pseudomallei* K96243, and the data were visualized using the Artemis genome browser (5). *De novo* assembly of each genome was also generated using SPAdes genome assembler (Galaxy Version 3.12.0 + galaxy1) or BV-BRC (http://bv-brc.org). Sequencing data are available through GenBank (accession no. PRJNA1196838).

**TABLE 1:** Details of *B. pseudomallei* strains used in this study, MICs, and *penA* mutations^*b*^^,^^*c*^

Patient #	Year	Strain ID	Specimen type	No. of days after admission that specimen was collected	Sequence type (ST)	BMD MIC (mg/L)		Known or novel AAS mutation within, or near four Ambler’s motifs				Other AAS mutations	*−78A* mutation (G->A)	*penA* gene duplication and amplification (GDA)	Copy number of *penA* (dd PCR)
						CAZ	AMC	^70^SxxK^73^	^130^SDN^132^	^166^ExxLN^170^	^234^KTG^236^				
1	1987	316a	Blood	6	17	2	2/1	−	−	−	−	−	−	−	n/a
		316c	Blood	24	17	**32**	2/1	−	−	P167S	−	−	−	−	n/a
2	1988	365a	Urine	2	34	**32**	**16/8**	−	−	−	−	−	+	−	n/a
		365c	Blood	3	34	2	2/1	−	−	−	−	−	−	−	n/a
3	1988	402a	Blood	0	60	2	1/0.5	−	−	−	−	−	−	−	n/a
		402g	Blood	9	60	**32**	1/0.5	−	−	P167S	−	−	−	−	n/a
		402h	Throat swab	9	60	**32**	1/0.5	−	−	P167S	−	−	−	−	n/a
4	1988	405a	Blood	11	33	**16**	**16/8**	−	−	−	−	−	−	+	1.9966
5	1989	490b	Sputum	2	10	2	2/1	−	−	−	−	−	−	−	n/a
		490f	Sputum	24	10	**64**	2/1	−	−	**A172T^*a*^**	−	−	−	+	2.9686
6	1989	533a	Sputum	4	17	4	4/2	−	−	−	−	−	−	+	1.6071
		533eii	Sputum	28	17	**>128**	4/2	−	−	P167S	−	−	−	+	1.8246
7	1989	577a	Throat swab	1	708	2	4/2	S72F	−	−	−	−	−	−	n/a
		577b	Blood	8	708	2	**64/32**	S72F	−	−	−	−	+	−	n/a
		577ci	Sputum	20	708	**>128**	**16/8**	C69Y, S72F	−	−	−	−	+	−	n/a
		577cii	Sputum	20	708	2	**>64/32**	S72F	−	−	−	−	−	+	8.9028
		577d	Sputum	32	708	2	**>64/32**	S72F	−	−	−	−	−	+	9.1499
8	1991	858ai	Blood	3	56	2	2/1	−	−	−	−	I139M, T147A	−	−	n/a
		858g	Blood	20	56	**32**	2/1	−	−	P167S	−	I139M, T147A	−	−	n/a
		858d	Synovial fluid	27	56	**32**	2/1	−	−	P167S	−	I139M, T147A	−	−	n/a
		858fi	Blood	27	56	**32**	2/1	−	−	P167S	−	I139M, T147A	−	−	n/a
9	1992	942a	Blood	0	17	2	2/1	−	−	−	−	−	−	−	n/a
		942dii	Surface swab	6	17	**32**	**16/8**	−	−	−	−	−	+	+	1.5720
10	1992	956a	Blood	1	54	2	2/1	−	−	−	−	I139M, T147A	−	−	n/a
		956c	Blood	15	54	**32**	2/1	−	−	P167S	−	I139M, T147A	−	−	n/a
11	1992	975a	Sputum	0	873	1	2/1	−	−	−	−	T147A	−	−	n/a
		975ci	Sputum	13	873	**32**	2/1	−	−	−	−	T147A	−	+	6.9819
		975d	Sputum	17	873	**32**	2/1	−	−	P167S	−	T147A	−	−	n/a
12	1992	979a	Throat swab	0	60	2	2/1	−	−	−	−	−		−	n/a
		979bi	Sputum	7	60	**16**	8/4	−	−	−	−	−	+	−	n/a
		979bii	Tracheal suction	31	60	**>128**	8/4	−	−	P167S	−	−	+	−	n/a
13	1992	984a	Sputum	0	70	1	2/1	−	−	−	−	T147A	−	−	n/a
		984d	Sputum	6	70	**16**	8/4	−	−	−	−	T147A	+	−	n/a
		984di	Sputum	6	70	**16**	8/4	−	−	−	−	T147A	+	−	n/a
14	1992	995a	Parotid pus	0	174	2	1/0.5	−	−	−	−	I139M, T147A	−	−	n/a
		995e	Parotid pus	21	309	**16**	**16/8**	−	−	−	−	−	+	−	n/a
15	1992	1005a	Sputum	0	198	2	4/2	−	−	−	−	−	−	−	n/a
		1005d	Sputum	12	198	**16**	**32/16**	−	−	−	−	−	+	−	n/a
16	1998	2085a	Blood	1	167	0.5	2/1	−	−	−	−	T147A	−	−	n/a
		2085g	Blood	29	167	**32**	8/4	−	−	−	−	T147A	+	−	n/a
17	1999	2374a	Sputum	1	174	4	4/2	−	−	−	−	I139M, T147A	−	−	n/a
		2374b	Sputum	3	304	4	**32/16**	S72F	−	−	−	−	−	−	n/a
18	1999	2381a	Throat swab	9	1646	1	2/1	−	−	−	−	P145L, T147A	−	−	n/a
		2381c	Sputum	18	4	**16**	8/4	−	−	−	−	T147A	−	+	5.0906
		2381d	Throat Swab	22	304	1	**32/16**	S72F	−	−	−	−	−	−	n/a
19	2001	2690a	Sputum	18	1464	**64**	2/1	−	−	−	D240G	T147A	+	−	n/a
20	2003	3013a	Sputum	1	874	1	2/1	−	−	−	−	−	−	−	n/a
		3013c	Sputum	19	874	**32**	8/4	−	−	−	−	−	−	+	4.4158
21	2006	3964b	Blood	1	671	2	2/1	−	−	P167S	−	I139M, T147A	−	−	n/a
		3964c	Tracheal suction	19	671	**32**	2/1	−	−	P167S	−	I139M, T147A	−	−	n/a
		3964d	Blood	19	671	**32**	2/1	−	−	P167S	−	I139M, T147A	−	−	n/a
22	2006	4095a	Sputum	1	1465	2	2/1	−	−	-−	−	−	−	−	n/a
		4095c	Pleural fluid	23	1465	**32**	**16/8**	−	−	−	−	−	−	+	6.1034
23	2007	4226a	Sputum	1	306	**32**	8/4	−	−	−	−	−	−	+	6.3249
		4226b	Throat swab	1	306	**16**	**8/4**	−	−	−	−	−	+	−	n/a
		4226c	Sputum	11	306	**16**	**8/4**	−	−	−	−	−	+	−	n/a
24	2007	4609a	Sputum	1	17	1	1/0.5	−	−	−	−		−	−	n/a
		4609e	Tracheal suction	22	17	**32**	**16/8**	−	−	−	−		−	−	n/a

^*a*^ A novel AAS mutation characterized in this study; bold indicates intermediate - resistance or resistance MIC levels. C69Y (Cysteine to Tyrosine), S72F (Serine to Phenylalanine), P167S (Proline to Serine), A172T (Alanine to Threonine), and D240G (Aspartic to Glycine); the numbers are assigned according to the Ambler’s numbering scheme. Strain information and Etest results were previously documented in reference 1 and are shown alongside BMD results in Table S1 for comparison.

^*b*^ "+" and "−" indicate the presence and absence, respectively, of the specific mutation or genetic feature listed in each column.

^*c*^ n/a, not applicable.

**Fig 1:**
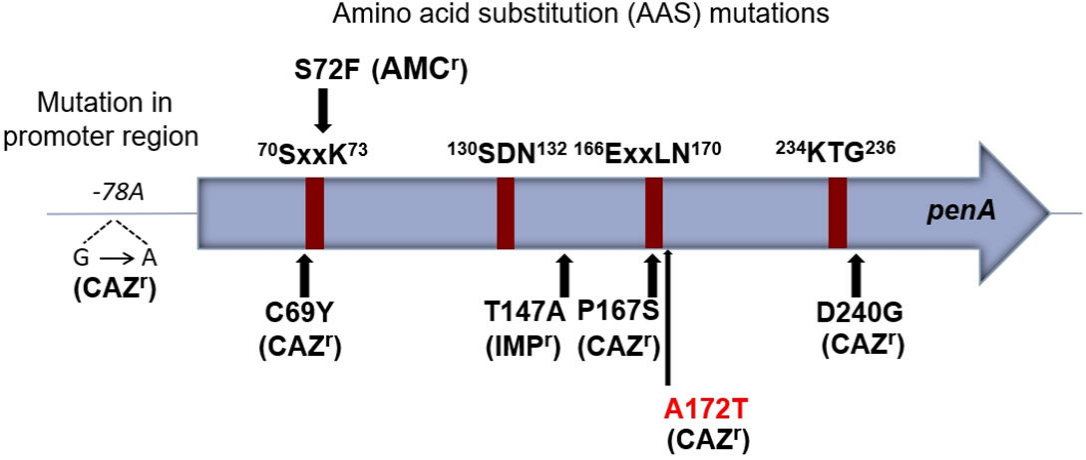
AAS mutations in *penA* and in a promoter region known to be associated with amoxiclav resistance (AMC^r^), imipenem resistance (IMP^r^), or ceftazidime resistance (CAZ^r^). A172T is a novel AAS mutation responsible for CAZ resistance identified and characterized in this study.

Our findings revealed a complex landscape of genetic alterations associated with CAZ and AMC resistance. We identified eight distinct amino acid substitutions (AASs) in the *penA* gene, which encodes a class A β-lactamase enzyme known to confer resistance to β-lactam antibiotics, especially CAZ and AMC based on the BMD results (Table 1). Among these substitutions, five were known to be responsible for CAZ, AMC, or imipenem (IMP) resistance (Fig. 1) (6–9), while three were novel AAS mutations, I139M, P145L, and A172T. We observed that P174L, the most recently reported AAS mutation associated with CAZ resistance in Hainan, China (9), was not present in our strains. Among the novel mutations, only the A172T (Alanine to Threonine) substitution near ^166^ExxLN^170^, one of Ambler’s motifs (10) in *B. pseudomallei* strain 490f showed a particularly strong association with increased MICs for CAZ. This mutation was absent in the CAZ-susceptible strain 490b, which was isolated three weeks earlier from the same patient during the hospitalization. To confirm whether this mutation was responsible for the increased CAZ MIC, allelic exchange mutagenesis was performed to introduce the A172T mutant in *penA* gene of *B. pseudomallei* Bp82, a biosafe and CAZ-susceptible strain (11, 12); see Text S1. The resulting mutant exhibited a 16-fold increase in CAZ MIC, confirming the mutation’s critical role in resistance.

**Fig 2:**
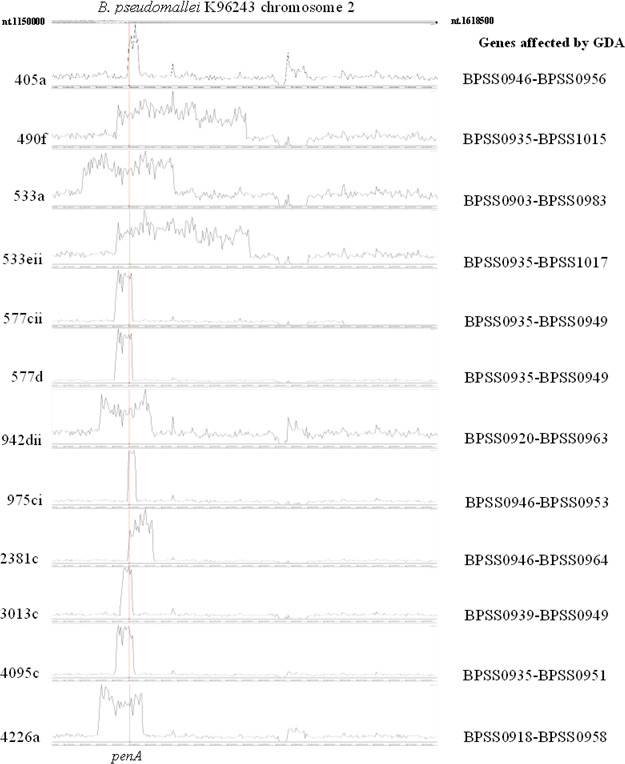
The GDA of the *penA* gene, highlighting affected genomic regions observed from mapping Illumina short reads of 12 CAZ-resistant *B. pseudomallei* strains against chromosome 2 of the reference *B. pseudomallei* K96243. Amplified regions, indicated by peaks in read depth, with the *penA* highlighted in red.

**Fig 3:**
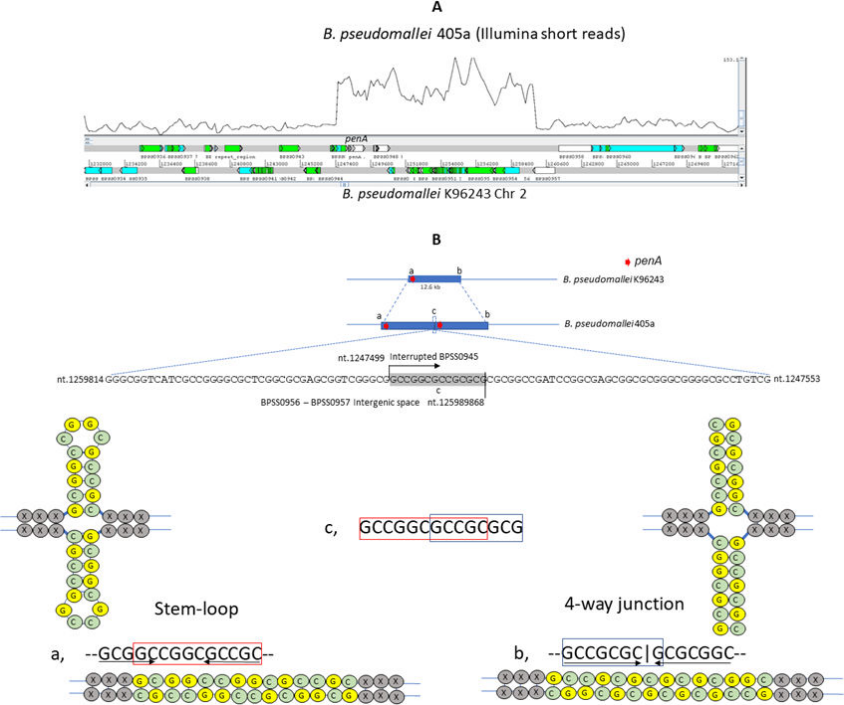
An example of GDA of *penA* mediated by PGCRRS. (**A**) Genomic region containing *penA* and its neighboring genes, observed by mapping Illumina short reads from *B. pseudomallei* 405a against chromosome 2 of the reference strain K96243. (**B**) Hypothetical model illustrating GDA mediated by the recombination of the PGCRRS features, including: (a) a cruciform stem-loop structure, (b) a cruciform four-way junction structure, and (c) a 14 bp sequence resulting from the recombination between (a) and (b). Note: The genome coordinates shown correspond to those of chromosome 2 of *B. pseudomallei* K96243.

In addition to AAS mutations, we identified a promoter mutation, specifically the −*78A* mutation (13), of the *penA* gene in the CAZ-resistant *B. pseudomallei* strains isolated from 10 (41.6%) of the 24 patients. This mutation is known to enhance the expression of *penA*, thereby increasing the production of the β-lactamase enzyme and contributing to the observed resistance (13). Another major resistance mechanism identified was gene duplication and amplification (GDA) of the *penA* gene, observed in 12 CAZ-resistant strains from 10 (41.7%) of the 24 patients analyzed (Fig. 2). Digital droplet PCR assays (Bio-Rad QX200 Droplet Digital PCR System) using genomic DNA from *B. pseudomallei* K96243 as a single copy *penA* control confirmed that *penA* gene copies varied from two to nine among these strains, suggesting that *penA* GDA significantly increased resistance by enhancing β-lactamase enzyme production as previously reported by us (3). In most GDA events, the junction sequences, ranging from 5 to 16 bp in length and enriched in GC content, likely resulted from homologous recombination between cruciform structures, such as stem-loop cruciform or four-way junction cruciform sequences, found at both ends of the GDA region as exemplified by strain 405a (Fig. 3). We termed this genetic structure “Palindromic GC-Rich Repeat Sequences” (PGCRRS), as it appeared to mediate the homologous recombination of the GDA. We hypothesize that these cruciform structures induce replication stress in *B. pseudomallei* under CAZ selection, leading to double-strand breaks and complicating the DNA repair processes, thereby contributing to GDA. GDA also resulted in extra copies of variable genomic regions. For example, two copies of genes *BPSS0935*–*BPSS1017* were found in strain 533eii, while nine copies of genes between *BPSS0935* and *BPSS0949* were observed in strains 577cii and 577d (Fig. 2). To confirm the GDA, initially identified in Illumina assembly contigs, we utilized the Oxford Nanopore Technologies (ONT) sequencing platform, which offers longer reads to effectively cover the GDA’s junctions. This approach confirmed the junction sequences and genomic regions affected by the GDA in three selected strains: 490f, 533a, and 942dii (BioSample accession numbers SAMN45667634, SAMN45667635, and SAMN45667647, respectively). The GDA events in all 12 strains reported in this study are described in Table S1. The addition of ONT sequencing and the hybrid genomic assembling approach for strain 490f, as an example, are described in Text S2.

Additionally, we have observed significantly higher MICs for CAZ or AMC in strains that contained an AAS mutation in combination with the *−78A* promoter mutation and/or GDA. Notably, in strain 577ci, the presence of C69Y mutation occurring against the background of the S72F mutation within the Ambler’s motif ^70^SxxK^73^ in an earlier strain 577b shifted the resistance phenotype from AMC to CAZ. This finding suggests that modifications to *penA*’s active site may influence substrate specificity. Further investigation using the artificial intelligence-driven crystal structure analysis and molecular docking approach is warranted to elucidate the underlying mechanisms. However, in this current study, we were unable to determine the genetic or molecular basis of CAZ resistance in strain 4609e from patient #24. The *penA* and other CAZ resistance-associated genes, including penicillin-binding protein 3 (*BPSS1219*) (14) in this strain, were identical to those in the earlier CAZ-susceptible strain 4609a from the same patient. This observation underscores the need for additional research to identify alternative mechanisms contributing to CAZ resistance in such cases.

In addition to *penA*-mediated resistance, other contributors to β-lactam resistance in *B. pseudomallei*, though comparatively rare, include mutations in *amrR*, the regulatory gene of the AmrAB-OprA resistance-nodulation-cell division (RND) efflux pumps. These mutations have been associated with resistance to both aminoglycosides and β-lactams in clinical isolates (15), and sub-inhibitory antibiotic concentrations, resulting from inadequate dosing or poor adherence, may further induce *amrR*-mediated overexpression of AmrAB-OprA, contributing to MIC creep and treatment failure (16). Although efflux pump-related resistance mechanisms were not investigated among the isolates analyzed in this study, the potential role of sub-inhibitory antibiotic concentrations in driving resistance evolution warrants further investigation, particularly in endemic settings where dosing compliance may be variable. Another well-characterized mechanism involves the *penA* T147A substitution, which confers reduced susceptibility to carbapenems such as imipenem and meropenem (8). While these pathways reflect the genomic plasticity of *B. pseudomallei*, our findings highlight the dominant role of *penA* point mutations, promoter mutations, and GDA in driving CAZ resistance.

On another note, multi-locus sequence typing (MLST) analysis revealed that in most patients, initial isolates and resistant strains shared the same sequence types (STs), except in three patients (Table 1: patient #14, #17, and #18), the resistant strains had different STs from those initially identified at admission. This suggests that the resistant strains arose from distinct *B. pseudomallei* subpopulations that were not detected in the initial samples. Given the genetic diversity of *B. pseudomallei* in soil as previously described (17), it is possible that these patients were exposed to multiple strain genotypes. Genotyping of multiple isolates at admission warrants further investigation of strain diversity. Although our findings on these three patients support ST heterogeneity, it remains possible that resistance could have emerged within individual susceptible clones through sequential sub-inhibitory exposure to CAZ or AMC, mimicking suboptimal dosing or poor patient compliance. This process may promote mutations or gene amplification events like GDA of *penA*. Laboratory-based evolution experiments simulating this scenario would help confirm this hypothesis and may offer additional insights into resistance emergence mechanisms in clinical settings.

Unlike many gram-negative bacteria where AMR arises through horizontal gene transfer via plasmids, AMR in *B. pseudomallei* develops almost exclusively through chromosomal mutations, including point mutations, gene duplications, and regulatory changes. CAZ resistance in *B. pseudomallei* remains relatively uncommon, at initial presentation in northeast Thailand, reported at <1% (1), but has been increasingly reported, especially in patients with relapsed or prolonged infections (18–20). In conclusion, our findings provide critical insights into the genetic and molecular basis of CAZ resistance in *B. pseudomallei*. The identification of the novel A172T mutation and other previously known AAS mutations, the −*78A* promoter mutation, and the frequent occurrence of *penA* GDA events significantly advance our understanding of how this pathogen adapts under antibiotic pressure. Additionally, *penA*-mediated CAZ resistance appears to be increasing, as reported in multiple recent studies (9, 19). These findings from us and others emphasize the importance of developing rapid diagnostic assays that can detect these genetic alterations, guiding more effective treatment decisions in clinical practice. While phenotypic AST, such as BMD or disk diffusion, remains the gold standard in clinical microbiology, its turnaround often delays appropriate treatment decisions. A potential rapid diagnostic assay could employ multiplex PCR or CRISPR-based detection to identify known resistance-conferring mutations such as *penA* point mutations (e.g., C69Y, S72F, P167S, A172T, P174L, D240G), the −*78A* promoter mutation, and the presence of *penA* duplication.

While rapid molecular assays targeting known resistance mechanisms may offer earlier insights in selected cases, their overall clinical utility remains limited. Resistance in *B. pseudomallei* primary isolates is rare, and when it emerges during therapy, it is often suspected based on poor clinical response. Moreover, the technical challenges of developing multiplex diagnostics and the inability to detect unknown or regulatory mutations underscore the continued importance of phenotypic testing. Nonetheless, such tools may serve as useful adjuncts in endemic areas with high relapse rates, where earlier resistance detection could support more timely treatment adjustments.

## SUPPLEMENTAL MATERIAL

Supplemental material aac.00220-25-s0001.docx Includes Etest methodology.

Supplemental material 2 aac.00220-25-s0002.docx Methodology of Oxford Nanopore Technology and Illumina sequencing techniques and genomic assembly.

Table S1 aac.00220-25-s0003.xlsx Details of bacterial strain, GenBank accession, MLST, Etest, GDA, and penA mutations.

ASM does not own the copyrights to Supplemental Material that may be linked to, or accessed through, an article. The authors have granted ASM a non-exclusive, world-wide license to publish the Supplemental Material files. Please contact the corresponding author directly for reuse.
