# Supplementary material for: Genomic insights into ceftazidime resistance in Burkholderia pseudomallei: discovery of A172T mutation and palindromic GC-rich repeat sequences facilitating penA duplication and amplification
Source: Antimicrob Agents Chemother. 2025 Jul 21;69(8):e00220-25. doi: 10.1128/aac.00220-25 (PMC12326997; doi:10.1128/aac.00220-25)
Supplement: Supplemental material — Includes Etest methodology. [file aac.00220-25-s0001.docx]

**Supplemental Text 1:**

To generate a single base A172T substitution in PenA (BP1026B_II1037), we modified a technique from López CM et al., 2009 [1]. Briefly, approximately 650 base pairs upstream and 700 base pairs downstream of the target nucleotide were amplified using the following four primers in a single PCR reaction. These primers included:

penA A172T 5'_fwd: 5’-GCTGAACACGACGCTGCCCGGCGACGAG-3’,

penA A172T 5'_rev: 5’-CGGGCAGCGTCGTGTTCAGCTCAGGCTC-3’,

penA A172T 3'_fwd: 5’-GCTGAACACGACGCTGCCCGGCGACGAG-3’, and

penA A172T 3'_rev: 5’-GGGATAACAGGGTAATCCCGATACCGGCATCGTTTCGCTGCG-3’.

The PCR fragments were then gel purified using the Zymoclean Gel DNA Recovery Kit. To construct the pExKm5-A172T PenA plasmid, the pExKm5 plasmid was digested using *EcoRI*-HF and *NotI* (New England Biolabs, NEB) and gel purified alongside the PCR products. Note: The plasmid pExKm5 was kindly provided by Dr. Schweizer at the University of Florida. The digested plasmid and PCR fragments were assembled using the NEBuilder HiFi DNA Assembly Master Mix (New England Biolabs, NEB), following the manufacturer’s protocol. The assembled plasmid was then transformed into DH5α *E. coli* on LB plates supplemented with 50 μg/ml of kanamycin (Km) and 5-bromo-4-chloro-3-indolyl-β-d-galactopyranoside (X-Gal). White colonies were selected for plasmid amplification. *B. pseudomallei* Bp82 electrocompetent cells were prepared in-house by washing three times with 300 mM sucrose, as described by Choi KH et al., 2005 [2]. Two microliters of purified plasmid were electroporated into 100 μL of competent cells using a 0.2 mm cuvette (Bio-Rad) at 2500 V, 200 Ω. The transformed cells were recovered in LB broth for 2 hours with slow shaking, then plated on LB plates containing 250 μg/mL Km and 50 μg/mL X-Gluc for 48 hours. A blue Km-resistant colony was picked, and the merodiploid was resolved on 15% sucrose YT agar supplemented with 0.8 μg/mL adenine for 48 hours. White colonies were selected on LB adenine agar supplemented with 32 μg/ml ceftazidime. The resistant colonies were then screened by PCR using penA A172T 5'_fwd and penA A172T 3'_rev primers, followed by amplicon sequencing to confirm the mutation. In addition, whole genome sequencing by Illumina was used to confirm the presence of A172T allele in *penA* of the mutant CAY2 (BioSample# SAMN46427263).

Minimal inhibitory concentrations (MICs) of ceftazidime (CAZ) were determined for *B. pseudomallei* strains CAY2 (PCR-positive mutant) and Bp82 (parental strain) using a test strip (Liofilchem^TM^ MTS^TM^ Ceftazidime; 0.016-256 µg/mL). Briefly, overnight cultures grown on LB agar supplemented with 80 mg/L adenine were used to prepare bacterial suspensions in sterile normal saline. The turbidity of each suspension was adjusted to a 0.5 McFarland standard, corresponding to an optical density at 600 nm (OD₆₀₀) of 0.08–0.10. Cation-Adjusted Mueller-Hinton Agar (CAMHA; 20 mL) was poured into sterile plastic Petri dishes (100 mm × 15 mm) and allowed to solidify. The bacterial suspension was uniformly spread across the surface using a sterile cotton swab while rotating the plate on a plate rotator to ensure even coverage. After letting the surface moisture absorb (~15 minutes), a CAZ test strip was placed at the center of the agar plate. Plates were incubated at 35°C for 24 hours. After incubation, the MIC was recorded as the concentration at which the ellipse of inhibition intersected the scale on the test strip (see below).


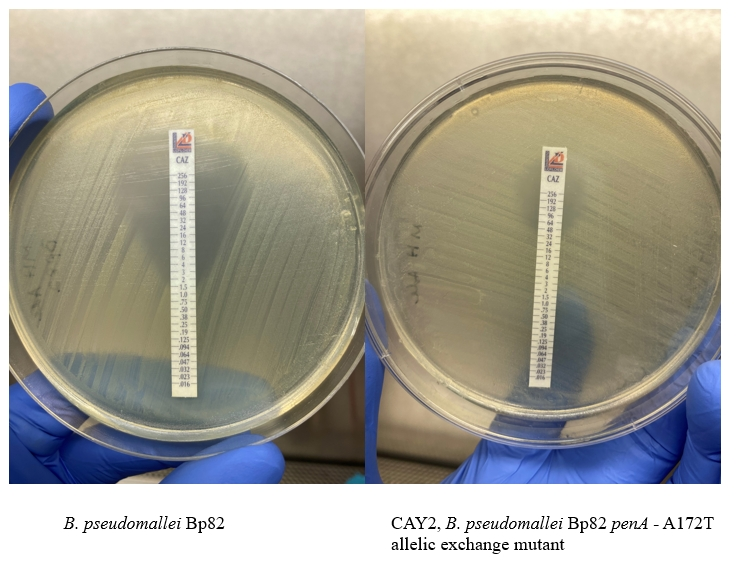


The MIC for the mutant strain CAY2 was determined to be 32 µg/mL, compared to 1.5–2 µg/mL for the parent strain Bp82, indicating a substantial increase in ceftazidime resistance conferred by the A172T mutation in *penA.*

In parallel, the broth microdilution (BMD) method was performed according to Clinical and Laboratory Standards Institute (CLSI) guideline (M07, 11^th^ ed.), Using this method, the MICs for strains CAY2 and Bp82 were confirmed to be 32 µg/mL and 2 µg/mL, respectively.

**Reference**

1. López, C.M.; Rholl, D.A.; Trunck, L.A.; Schweizer, H.P. Versatile Dual-Technology System for Markerless Allele Replacement in Burkholderia Pseudomallei. *Appl. Environ. Microbiol.* **2009**, *75*, 6496–6503, doi:10.1128/AEM.01669-09.

2. Choi, K.-H.; Kumar, A.; Schweizer, H.P. A 10-Min Method for Preparation of Highly Electrocompetent Pseudomonas Aeruginosa Cells: Application for DNA Fragment Transfer between Chromosomes and Plasmid Transformation. *J. Microbiol. Methods* **2006**, *64*, 391–397, doi:10.1016/j.mimet.2005.06.001.

**Supplemental Text 2: Etest procedure for MIC determination**

To determine the minimal inhibitory concentration (MIC) of ceftazidime (CAZ) against B. pseudomallei strains CAY2 and Bp82, an Etest was performed using a Liofilchem™ MTS™ ceftazidime strip (CAZ; concentration range 0.016–256 µg/mL). Bacterial cultures were prepared by suspending colonies of the overnight culture on LB agar supplemented with 80mg/L Adenine in sterile normal saline to achieve a turbidity equivalent to a 0.5 McFarland standard, corresponding to an OD₆₀₀ of 0.08–0.10.

Twenty milliliters of Cation-Adjusted Mueller-Hinton Agar (CAMHA) were poured into sterile 100 mm × 15 mm plastic Petri dishes and allowed to solidify. The prepared bacterial suspension was uniformly spread across the agar surface using a sterile cotton swab while rotating the plate on a plate rotator to ensure even inoculation. After allowing the surface moisture to absorb (~15 minutes), a ceftazidime Etest strip was applied to the center of the agar plate. The plates were then incubated at 35°C for 24 hours. Following incubation, the MIC was determined by reading the point at which the ellipse of inhibition intersected the test strip scale.

The MIC for the mutant strain CAY2 was determined to be 32 µg/mL, compared to 1.5–2 µg/mL for the parent strain Bp82, indicating a substantial increase in ceftazidime resistance conferred by the A172T mutation in penA.
