# Supplementary material for: Genomic insights into ceftazidime resistance in Burkholderia pseudomallei: discovery of A172T mutation and palindromic GC-rich repeat sequences facilitating penA duplication and amplification
Source: Antimicrob Agents Chemother. 2025 Jul 21;69(8):e00220-25. doi: 10.1128/aac.00220-25 (PMC12326997; doi:10.1128/aac.00220-25)
Supplement: Supplemental material 2 — Methodology of Oxford Nanopore Technology and Illumina sequencing techniques and genomic assembly. [file aac.00220-25-s0002.docx]

**Supplemental Text 2:**

The genome of *B. pseudomallei* strain 490f was sequenced using a hybrid approach combining long-read Oxford Nanopore Technologies (ONT) and short-read Illumina sequencing. Briefly, the bacteria were cultured in LB broth at 37^o^C with 250 rpm shaking overnight. DNA was extracted using the Promega’s Wizard® Genomic DNA Purification Kit according to the manufacturer’s instructions.

**Oxford Nanopore Sequencing:**
 ONT sequencing was conducted on the GridION platform using an R10.4.1 flow cell and the PCR-free ONT Ligation Sequencing Kit (SQK-NBD114.24) in combination with the NEBNext® Companion Module (E7180L). The sequencing yielded 157,168 reads with an average read length of 3,165 bp. Reads were filtered for a minimum length of 2,000 bp using SeqKit v2.4.0 [1], resulting in 69,268 reads with an average read length of 5,629 bp. Genome assembly was performed with Canu v2.2 [2], producing two circular chromosome scaffolds with an average coverage of 57.46x. Unless otherwise noted, default parameters were used for all software.

**Illumina Sequencing:**
 Illumina libraries were prepared using the Illumina DNA Prep Kit and NEBNext® Multiplex Oligos for Illumina (dual-indexed primers), targeting a 280-bp insert size. Paired-end sequencing (2 × 151 bp) was performed on the Illumina NextSeq platform, generating 18,124,202 reads. Quality control and adapter trimming were performed with bcl-convert1 v4.2.4. Reads were aligned to the ONT-derived genome scaffolds using Bowtie2 v2.4.2 [3]. Pilon v1.23 [4] was used to polish the draft assembly, resulting in a final average coverage of 749x.

**Repeat sequence correction:**

Initial analysis of the polished sequences in CLC Genomics Workbench v20.0.4 revealed a threefold increase in read coverage of the long reads at coordinates 2.1–2.3 Mb on chromosome 2. A 150-kb region from this area was extracted and manually re-aligned to improve mapping accuracy. To resolve this, ambiguous bases (N) were added upstream and downstream of the sequence, followed by re-mapping. There was a subset of three identical repeat sequences, two of which had unique sequence endings that aligned upstream and downstream of the 150-kb region. The three repeat regions were then internally connected each other. These adjustments ensured proper assignment of the three repeat regions.

**References**

1. Shen W, Le S, Li Y, Hu F. 2016. SeqKit: A Cross-Platform and Ultrafast Toolkit for FASTA/Q File Manipulation. PLoS One 11:e0163962.

2. Koren S, Walenz BP, Berlin K, Miller JR, Bergman NH, Phillippy AM. 2017. Canu: scalable and accurate long-read assembly via adaptive k-mer weighting and repeat separation. Genome Res 27:722–736.

3. Langmead B, Salzberg SL. 2012. Fast gapped-read alignment with Bowtie 2. Nat Methods 9:357–359.

4. Walker BJ, Abeel T, Shea T, Priest M, Abouelliel A, Sakthikumar S, Cuomo CA, Zeng Q, Wortman J, Young SK, Earl AM. 2014. Pilon: An Integrated Tool for Comprehensive Microbial Variant Detection and Genome Assembly Improvement. PLoS One 9:1–14.
